# Hybrid Transcriptional Regulators for the Screening of Target DNA Motifs in Organohalide-Respiring Bacteria

**DOI:** 10.3389/fmicb.2020.00310

**Published:** 2020-03-03

**Authors:** Mathilde Stéphanie Willemin, Marie Vingerhoets, Christof Holliger, Julien Maillard

**Affiliations:** Laboratory for Environmental Biotechnology, School of Architecture, Civil and Environmental Engineering, Ecole Polytechnique Fédérale de Lausanne (EPFL), Lausanne, Switzerland

**Keywords:** organohalide respiration, transcriptional regulators, CRP/FNR superfamily, reductive dehalogenase, hybrid proteins, RdhK, *Desulfitobacterium*

## Abstract

The bioremediation of persistent organohalide molecules under anoxic conditions mostly relies on the bacterial process called organohalide respiration (OHR). Organohalide-respiring bacteria (OHRB) are phylogenetically diverse anaerobic bacteria that share the capacity to use organohalides as terminal electron acceptors in an energy-conserving process. The reductive dehalogenase (*rdh*) gene clusters encode for proteins specialized in the respiration of one or a limited number of organohalides. One particular OHRB may harbor up to several dozens of *rdh* gene clusters suggesting a wide potential for bioremediation. To avoid wasting energy in producing unnecessary proteins, *rdh* gene clusters often include a transcriptional regulator. In organohalide-respiring *Firmicutes*, RdhK is a dedicated transcriptional regulator of OHR and represents a subfamily of proteins among the CRP/FNR superfamily of regulators. RdhK proteins are composed of an effector-binding domain (EBD) which recognizes a given organohalide and subsequently controls the interaction of its C-terminal DNA-binding domain (DBD) with a DNA motif (referred to as dehalobox, or DB) located in the promoter region of the target *rdh* genes. The two binding partners (i.e. an organohalide molecule and a DB sequence) of RdhK proteins are interdependent which impairs the exploration of OHR regulatory networks. Here, we propose a strategy relying on hybrid proteins to efficiently screen the DNA target of a single RdhK protein without prior knowledge on its effector. To demonstrate the potential of the method, two hybrids with alternative fusion points were designed based on RdhK6 EBD and RdhK1 DBD from *Desulfitobacterium hafniense*. Electrophoretic mobility shift assay was performed with purified hybrids along with the parental proteins and their binding properties were further tested *in vivo* through a β-galactosidase reporter assay. Along with revealing new RdhK6 features, we show that both hybrids resulted in active regulatory proteins with distinct binding patterns. While Hybrid A was less specific for the DNA motif, Hybrid B successfully mimicked the binding behavior of the parental proteins and thus represents a promising template for the design of new RdhK hybrids to screen yet uncharacterized RdhK proteins and also possibly other members of the CRP/FNR superfamily.

## Introduction

Organohalide-respiring bacteria (OHRB) are capable of conserving energy by using organohalide molecules as terminal electron acceptors (Adrian and Löffler, 2016). This energy metabolism occurs in strict anaerobes belonging to three different phyla (*Firmicutes*, *Proteobacteria*, and *Chloroflexi*) through a family of enzymes called reductive dehalogenases (RDases). These enzymes are generally encoded in *rdh* gene clusters composed by a minimum of two genes corresponding to the reductive dehalogenase catalytic subunit (*rdhA*) and its putative membrane anchor (*rdhB*) (Hug and Edwards, 2013). The genomes of some OHRB contain up to three dozens of *rdh* gene clusters (McMurdie et al., 2009), which suggests a broader dehalogenation potential (of natural and anthropogenic organohalides) than recognized today (Atashgahi et al., 2018). However, considering that each gene cluster is dedicated to the respiration of one or a limited number of organohalides, there is a need for tight regulation of the genes involved in the metabolism of the corresponding compounds.

Often, *rdhAB* genes are surrounded by a number of accessory genes encoding proteins of different functions (Kruse et al., 2015, 2016; Maillard and Willemin, 2019). Three main types of transcriptional regulators are distributed among OHRB mostly following their phylogeny [for a recent review, see Maillard and Willemin (2019)]. The major family of transcriptional regulators that emerged in the *Firmicutes* OHRB is based on RdhK, a subfamily belonging to the CRP/FNR superfamily (Kruse et al., 2016; Maillard and Willemin, 2019). Generally, RdhK proteins harbor an N-terminal effector-binding domain (EBD) linked via a central α-helix region to a C-terminal helix-turn-helix DNA-binding domain (DBD). Typically, the recognition of one specific organohalide molecule by the EBD sterically controls the interaction of the DBD with a specific DNA motif, called dehalobox [or DB, as defined previously (Gábor et al., 2006)], located in the promoter region of the target *rdh* genes (Maillard and Willemin, 2019), thus forming a ternary complex. Interaction of RdhK proteins with the promoter recruits the RNA polymerase which will proceed with transcription of the downstream genes. Only a few studies have reported the diversity of RdhK proteins (Kim et al., 2012; Rupakula et al., 2013; Kruse et al., 2015), among which only a few representatives have been characterized so far. The large majority of the available information and the mechanistic model come from the study of CprK from *Desulfitobacterium dehalogenans* strain JW/IU-DC1 (Smidt et al., 2000; Pop et al., 2004, 2006; Joyce et al., 2006; Gupta and Ragsdale, 2008) and its homolog, CprK1, from *Desulfitobacterium hafniense* strain DCB-2 (Gábor et al., 2006, 2008; Joyce et al., 2006; Mazon et al., 2007; Levy et al., 2008; Kemp et al., 2013). More recently, CprK1 has been renamed RdhK6 to account for the overall RdhK diversity present in the genome of strain DCB-2 (Kim et al., 2012).

The RdhK6 encoding sequence is part of the chlorophenol reductive dehalogenase gene cluster in which three DB motifs have been detected (Gábor et al., 2006). Among them, RdhK6 has the strongest affinity for DB7 and this interaction is dependent on the presence of various chlorophenols with 3-chloro-4-hydroxyphenylacetic acid (Cl-OHPA) being considered as the strongest effector (Gábor et al., 2006, 2008). DB7 represents the paradigmatic dehalobox as it consists of 5-bp perfect inverted repeats (5′-TTAATacacATTAA-3′) centered at 41.5 bp upstream of the transcription start of the *cprBA* operon. The same positioning of the DNA motif in the promoter region has been reported for many other promoters targeted by other members of the CRP/FNR superfamily, like the one controlling the transcription of the *mglBAC* operon in *E. coli* (Weickertt and Adhya, 1993; Scott et al., 2003; Zheng et al., 2004). Extensive structural work on free and effector-bound RdhK6 proteins gave access to important residues involved in effector- and DNA-binding and in the global conformational change of RdhK6 dimers (note that several crystal structures have been also obtained for CprK from *D. dehalogenans*) (Joyce et al., 2006; Levy et al., 2008).

RdhK1, another transcriptional regulator of *D. hafniense* strain DCB-2 [originally named CprK4 (Gábor et al., 2008)] has been characterized to a lesser extent (Gábor et al., 2008). For RdhK1, *in vivo* reporter analysis has revealed DB8 (5′-TTAGTatacGCTAA-3′) as the target DNA motif (Gábor et al., 2008). However, two additional dehaloboxes (DB9 and DB10) also identified within the gene cluster encoding RdhK1 were not targeted by this regulatory protein. RdhK6 and RdhK1 proteins diverge in the nature of their effector molecules (preference for either *ortho*- or *meta-*substituted chlorinated phenols, respectively), and were therefore proposed to play a complementary role in *D. hafniense* (Gábor et al., 2008). However, RdhK1 showed a peculiar behavior with a significant level of DNA-binding activity in absence of any effector. Nevertheless, some organohalides like 3,5-dichlorophenol (3,5-DCP) have been shown to enhance RdhK1 binding to DB8 and were considered as effectors (Gábor et al., 2008).

Given the challenges of studying OHRB (slow growing, genetically intractable and strictly anaerobic bacteria) and the large diversity of *rdh* gene clusters encountered in most of the genomes, it is often difficult to associate a specific organohalide compound with a certain *rdh* gene cluster (Maphosa et al., 2010; Hug and Edwards, 2013; Maillard and Willemin, 2019). Therefore, the characterization of RdhK proteins represents a great opportunity to explore the diversity of *rdh* gene clusters as they usually target the promoter regions of the genes responsible for the respiration of their respective effector molecules.

For each new RdhK protein, the identification of its preferred effector and DNA motif represents an important challenge since both partners are interdependent in the formation of the ternary complex. In other words, the correct effector molecule is required to activate the RdhK protein of interest in order to screen for its DNA motif. Here we present a strategy where the complexity of the RdhK regulatory system is reduced by the use of hybrid proteins composed of the EBD of an already characterized RdhK protein (RdhK6) fused to the DBD of another member of the RdhK protein family. The advantage of this approach is to decouple the screening procedure for DNA motifs from the screening of the effector molecules. The present study aims at developing and validating the application of RdhK hybrid proteins for the screening of DNA motifs. To this respect, two different RdhK hybrid proteins composed of the EBD of RdhK6 (RdhK6_E_) that is activated by Cl-OHPA, and the DBD of RdhK1 (RdhK1_D_) that targets DB8, were designed and their respective binding properties were evaluated. Two versions of the RdhK6_E_-RdhK1_D_ hybrid (in short RdhK6_E_1_D_), which differed in the position of the fusion site between the two domains, were tested both *in vitro* and *in vivo* for binding to selected organohalides and DNA motifs, and were compared to the results obtained with the native parental proteins.

## Materials and Methods

### Bacterial Strains and Growth Conditions

For protein production, *Escherichia coli* strain BL21 (DE3) carrying the expression plasmid of interest was cultivated overnight in 3 mL lysogeny broth (LB) (Bertani, 1951) supplemented with kanamycin (30 μg/mL). The pre-culture was used to inoculate 500 mL of fresh medium following a 1:100 dilution. Large cultures were incubated at 37°C under agitation at 160 rpm until reaching approximately 0.6 of optical density at 600 nm (OD_600_). At that stage, to limit the formation of inclusion bodies, protein production was induced at 16°C by adding 0.1 mM of isopropyl β-D-1-thiogalactopyranoside (IPTG) and incubation was carried out for 3 h. After induction, cells were collected (7′000 × *g*, 4°C, and 15 min) and washed once with heparin binding buffer (see below for details) and the biomass was either stored at −80°C or directly used for protein purification.

In the context of the *in vivo* β-galactosidase reporter assay, *E. coli* strain JM109 (DE3) carrying both the plasmid for RdhK protein production and the reporter plasmid were cultivated in 3 mL M9 medium (42 mM Na_2_HPO_4_, 22 mM KH_2_PO_4_, 8.5 mM NaCl, 18.7 mM NH_4_Cl supplemented with 2 mM MgSO_4_, 0.1 mM CaCl_2_ and 0.2% glucose) with addition of kanamycin (30 μg/mL) and erythromycin (200 μg/mL) at 37°C for approximately 24 h. This culture served as pre-culture for the inoculation of 20 mL cultures at a dilution of 1:100. Growth was carried out at 37°C until reaching an OD_600_ value between 0.3 and 0.6. At this point, protein production was induced at 20°C by adding 0.1 mM IPTG. When appropriate, 0.1 mM of chlorinated compounds (from a 100 mM stock solution in ddH_2_O) was added at the same point. Cells were then incubated for 12 h at 20°C and 200 rpm before applying the β-galactosidase activity assay (see below).

### Plasmids Construction and DNA Manipulations

#### Cloning of *rdhK* Expression Plasmids

**FIGURE 1:**
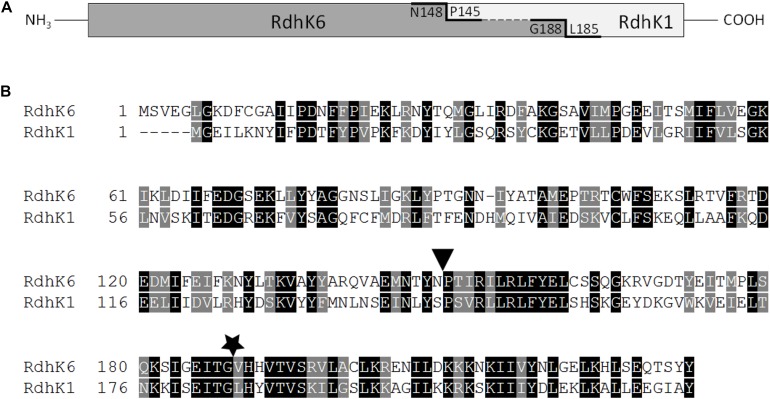
Design of RdhK hybrid proteins. **(A)** Schematic representation of fusion sites. Hybrid A comprises the effector-binding domain of RdhK6 (dark gray, amino acids 1–148) and the DNA-binding domain of RdhK1 (light gray, amino acids 145–228). Hybrid B was fused further down along the RdhK6 sequence after residue G188 of RdhK6 with amino acids 185-228 of RdhK1. The residues at the fusion sites are indicated. **(B)** Sequence alignment of both parental RdhK proteins from *D. hafniense* strain DCB-2. The fusion points of both hybrid proteins are indicated by a triangle for Hybrid A and by a star for Hybrid B. The alignment was obtained with ClustalX2.0 and illustrated with Boxshade.

All primers used in this study are given in Supplementary Table 1, while plasmids are described in Table 1. The sequence encoding *rdhK1* was cloned into the expression plasmid pET24d after PCR amplification (with primers MW031 and MW032) from genomic DNA of *Desulfitobacterium hafniense* strain DCB-2, resulting in the plasmid pMW021. The plasmid pWUR176 displaying the *rdhK6* gene of strain DCB-2 was obtained from Hauke Smidt (Wageningen University, Netherlands). An *E. coli* codon-optimized version of the sequence encoding RdhK Hybrid A (RdhK6_1__–__148_-RdhK1_145__–__228_, see Figure 1) was first amplified using primers RdhK61A-F/R from a plasmid produced by Eurofins Scientific AG (Schönenwerd, Switzerland) and was inserted in pET24d resulting in plasmid pRDHK61A. The sequence encoding RdhK Hybrid B (RdhK6_1__–__188_-RdhK1_185__–__228_, Figure 1) was obtained by fusion PCR. The sequences encoding the two protein domains were PCR amplified separately from genomic DNA of strain DCB-2 using primers MW033/MW034 and MW035/MW036, and fused together in a second PCR reaction (for details, see Supplementary Information). The resulting final sequence of 696 bp was cloned into linearized pET24d. The resulting plasmid was named pMW019.

**TABLE 1:** List of plasmids used in the study.

**Plasmid**	**Description**	**References**
pWUR176	pET24d plasmid for the expression of RdhK6	Gábor et al., 2006
pMW021	pET24d plasmid for the expression of RdhK1	This study
pRDHK61A	pET24d plasmid for the expression of RdhK hybrid A	This study
pMW019	pET24d plasmid for the expression of RdhK hybrid B	This study
pWUR166	pAK80 plasmid for promoter fusion to *lacLM* genes	Gábor et al., 2006
pMW032	pAK80 plasmid with DB07 containing original promoter	This study
pMW033	pAK80 plasmid with DB08 containing promoter	This study
pMW034	pAK80 plasmid with noDB containing promoter	This study

#### Design and Construction of the DB Chassis for *in vitro* Analysis

For each DB of interest, a 79-bp oligonucleotide was obtained from Microsynth AG (Balgach, Switzerland). These oligonucleotides were designed as to replace the CRP-binding motif of *E. coli mgl* promoter by the 20-nt long DB motif (14-nt DB7, DB8 or a random and non-palindromic control sequence named noDB, flanked by 3 nt of the original DNA sequence) (Supplementary Figure 1). The resulting DNA fragments, *mgl*-DB7, *mgl*-DB8 or *mgl*-noDB, were amplified by PCR using the primers DBC-F and DBC-R targeting the 5′- and 3′-end of the P*_*mgl*_* sequence, purified with the Qiagen PCR Purification Kit and quantified using the NanoDrop ND1000 apparatus.

#### Cloning of Reporter Plasmids

All the reporter plasmids used in this study (pMW032, -033 and -034) were constructed based on pWUR166, which displays the DB7-containing promoter region of *cprBA* fused to the *lacLM* genes (Gábor et al., 2006). To generate the variants of the *cpr* promoter (P*_*cpr*_*) displaying alternative DB motifs, the sequence was divided in three distinct parts: P*_*cpr*_*-5′ (86 bp), P*_*cpr*_*-3′ (96 bp), both common to all promoters and flanking a central region of 50 bp harboring the DB of interest (20-nt sequences, as above for the DB chassis) (Supplementary Figure 2). First, the P*_*cpr*_*-5′ and -3′ regions were amplified separately using genomic DNA from *D. hafniense* strain DCB-2 with the primer pairs BG1743/MW060 and MW061/BG1704, respectively. The 3′-end of MW060 and MW061 primers was designed in order to hybridize with the first and last 5 nt of the central part, respectively. The PCR fusion was performed by mixing the flanking PCR products with synthetic oligonucleotides carrying the different DB motifs in a 1:1:1 ratio, and by PCR amplification with primers BG1743 and BG1704. The resulting 222-bp DNA fragments were inserted in the linearized pAK80 vector as described previously (Gábor et al., 2006).

### Protein Production and Purification

Biomass pellets from cells producing the protein of interest were resuspended in heparin binding buffer (50 mM sodium phosphate buffer, pH 7.2, 10 mM dithiothreitol (DTT) and 100 mM NaCl) supplemented with 1 × SIGMA*FAST* protease inhibitor cocktail (Merck, Zug, Switzerland) and few crystals of DNase I (Merck), at a ratio of 10 mL per g of cells (wet weight) and lysed through three rounds of French press at 1000 psi. The soluble fraction was recovered by centrifugation (12′000 × *g*, 4°C, and 15 min) and loaded on a 5-mL heparin column for affinity purification (HiTrap^TM^ Heparin HP affinity column, GE Healthcare, Glattbrugg, Switzerland) attached to an ÄKTAprime^TM^ apparatus (GE Healthcare). Proteins were eluted from the column using a gradient of NaCl (up to 1 M) with heparin elution buffer and fractions were analyzed by SDS-PAGE according to standard procedures. Protein fractions of interest were pooled and dialyzed overnight against a phosphate buffer (50 mM sodium phosphate buffer, pH 7.2, 10 mM NaCl and 1 mM DTT). To further increase protein purity, the dialyzed fractions were concentrated (Amicon^®^ Ultra 10 K cut-off, Merck) and purified through size-exclusion chromatography (Superose^TM^ 12, 10/300 GL, GE Healthcare) in SEC buffer (50 mM sodium phosphate buffer, pH 7.2, 100 mM NaCl and 1 mM DTT). Protein concentration was measured using the Qubit^TM^ Protein Assay kit (Fisher Scientific, Reinach, Switzerland). When needed, protein samples were further concentrated as described above.

### Electrophoretic Mobility Shift Assay

Aliquots of 100 ng of *mgl*-DB chassis DNA were mixed in a 15-μL reaction with 200 μM of chlorinated compound (from 2 mM aqueous solutions of Cl-OHPA or 3,5-DCP) and 2 μM of RdhK protein in 1 × EMSA reaction buffer (100 mM Tris–HCl, pH 8.5, 40% glycerol, 10 mM MgCl_2_, 500 mM NaCl, and 5 mM EDTA), which was freshly supplemented with 10 mM DTT. The reaction was incubated for 30 min at room temperature. After incubation, 1.5 μL of 10 × EMSA loading buffer (25% glycerol in 100 mM Tris–HCl, pH 8.5, supplemented with a few crystals of bromophenol blue) were added to the reaction and 15 μL of the mixture was loaded on 8% polyacrylamide gel (0.5 × Tris-borate-EDTA buffer (TBE) (45 mM Tris base, pH 8.3, 45 mM boric acid, 1 mM EDTA), 8% acrylamide-bisacrylamide 37.5:1, 1.25% glycerol, and 1 mM EDTA) that was run for 30 min in 0.5 × TBE buffer prior to load the samples. After running the gel at 100 V for approximately 90 min, the gel was transferred in a 0.5 × TBE solution supplemented with 2 μg/mL of ethidium bromide, and incubated for at least 30 min. UV signal was finally captured on the Universal Hood II Gel Doc System (Bio-Rad, Cressier, Switzerland), and quantified using the Image Lab Software (Bio-Rad).

### β-Galactosidase Activity Assay

Following a 12 h period of RdhK protein induction (see the section on bacterial growth), 12 mL of the cultures were collected and centrifuged for 10 min at 4500 × *g* and room temperature. In order to strengthen the β-galactosidase activity, the cells were concentrated 20-fold by resuspending the biomass pellets in 600 μL of growth medium. Aliquots of 100 μL of the cell suspensions were used to measure the cell density at OD_600_ and the remaining samples were supplemented with 25 μL of toluene for cell permeabilization. The mixtures were vortexed for 30 s at full power and left on ice for 15 min before transferring 350 μL into fresh tubes. Aliquots of 50 μL of permeabilized cells were added to tubes containing 450 μL of Z-buffer (per liter: 8.52 g Na_2_HPO_4_, 6.24 g NaH_2_PO_4_⋅2H_2_O, 0.75 g KCl, and 0.25 g MgSO_4_⋅7H_2_O) freshly supplemented with 0.07% β-mercaptoethanol. After 10 min of temperature equilibration at 28°C, 100 μL of *ortho*-nitrophenol-β-galactoside (ONPG, 4 mg/mL stock in Z-buffer) was added as substrate for the β-galactosidase. Reaction tubes were incubated at 28°C until the development of faint yellow color. At this point, reactions were stopped by the addition of 250 μL 1 M Na_2_CO_3_ and the incubation time was recorded. Absorbance was measured at 420 nm. Miller units were calculated according to the following formula, where *t* is the reaction time in min and *V* is the volume in mL of concentrated cell suspension used in the assay:

A⁢c⁢t⁢i⁢v⁢i⁢t⁢y⁢(M⁢i⁢l⁢l⁢e⁢r⁢u⁢n⁢i⁢t)=A420⁢x⁢ 103t⁢x⁢V⁢x⁢O⁢D600

Finally, linear regression based on least square fit method was used to evaluate the correlation between the presence of Cl-OHPA and an increase of β-galactosidase activity as well as its significance.

## Results

### Domain Definition for the Design of RdhK Hybrid Proteins

Two different RdhK hybrid proteins were designed based on ligand-free and ligand-bound RdhK6 (CprK1) structures (Levy et al., 2008), as shown in Figure 1A. RdhK6 domain boundaries have been defined as follows: the EBD containing the β-barrel effector-binding pocket ends with residue F107 and is separated from the DBD by the central α-helix region (S108-N148), which is partially affected by ligand binding. Consequently, the DBD begins with residue P149 (Levy et al., 2008). Although structural analysis has revealed that the EBD and DBD act relatively independently from each other, both intra- and intermolecular interactions have been observed between the two domains upon dimer formation and ligand recognition (Joyce et al., 2006; Mazon et al., 2007; Levy et al., 2008). To assess whether the domains as defined above can be completely decoupled, the EBD of RdhK6 and DBD of RdhK1 were fused at the residue N148 and P145 of the respective parent protein (see sequence alignment in Figure 1B), resulting in the first RdhK hybrid, named Hybrid A. In contrast, the two domains in Hybrid B were fused at the site corresponding to a conserved glycine residue in both parental proteins (G188 and G184 in RdhK6 and RdhK1, respectively). The corresponding C-terminal portion of RdhK6 (G188-Y232) is located after an amino acid stretch likely responsible for intramolecular inter-domain interactions. Moreover, residues strictly involved in DNA binding are located downstream of the conserved glycine (Levy et al., 2008).

**FIGURE 2:**
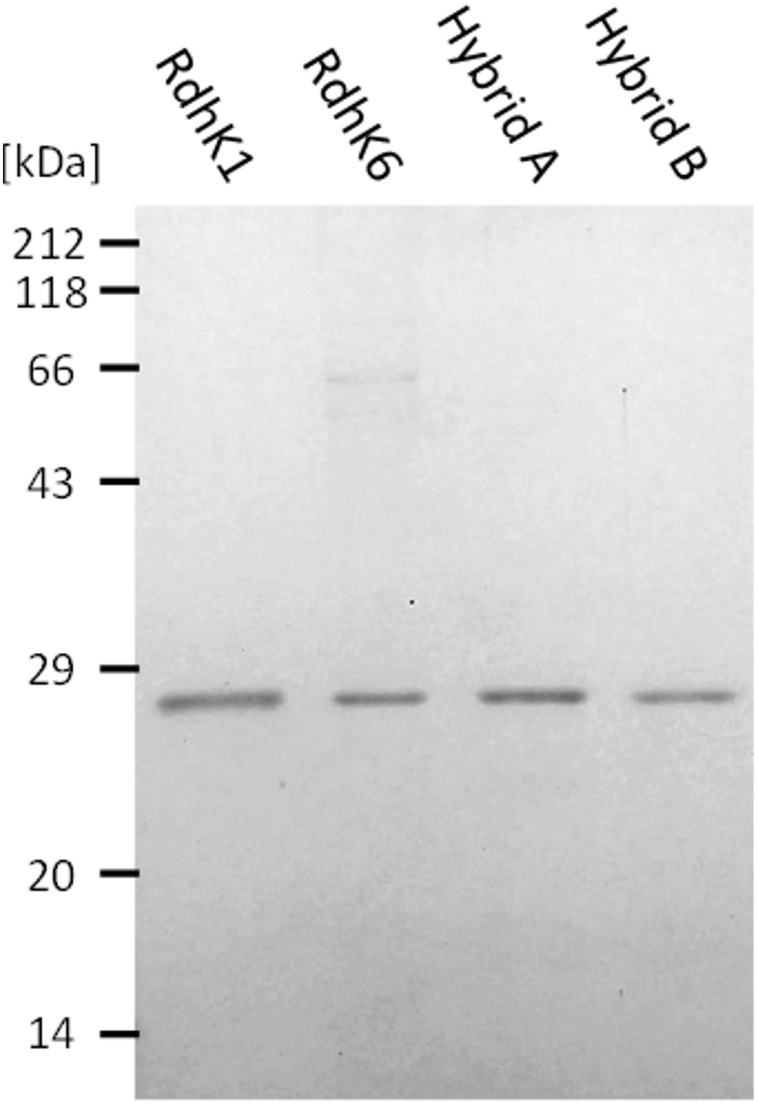
SDS-PAGE analysis of the four RdhK proteins used in EMSA. All four proteins were recombinantly produced in *E. coli* and purified by heparin affinity chromatography followed by size exclusion chromatography. The figure shows all four proteins loaded at the same concentration as they were used in EMSA. Samples were run in a 14% acrylamide gel which was stained with Coomassie G250 following standard procedures.

Hybrids A and B were recombinantly produced in and purified from *E. coli* along with RdhK6 and RdhK1. The binding activity of all four proteins, which were obtained in similar purity and comparable concentration (Figure 2), were first analyzed *in vitro* using EMSA.

### *In vitro* Characterization of RdhK Hybrid Proteins

#### Experimental Design and Controls

**FIGURE 3:**
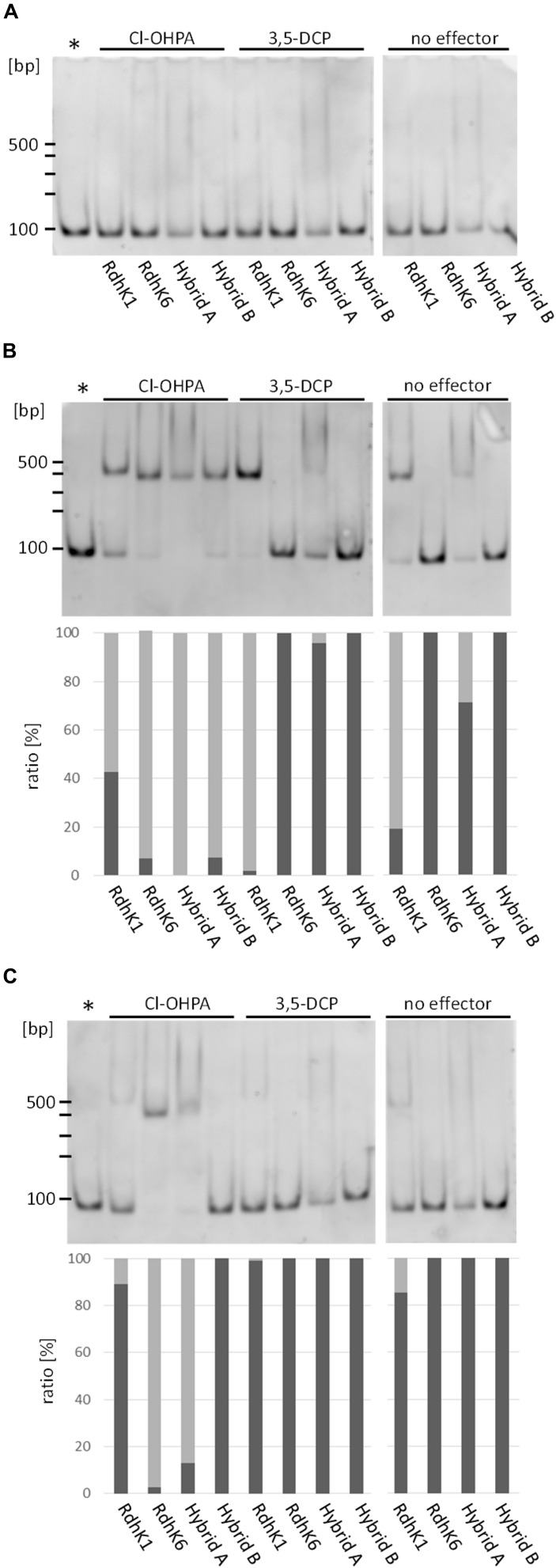
*In vitro* interactions of the RdhK parental and hybrid proteins with dehalobox DNA motifs. Electrophoretic mobility shift assay was performed with the four RdhK proteins (RdhK1, RdhK6, Hybrid A and B) and the following three DNA sequence: no DB **(A)**, DB8 **(B)** and DB7 **(C)**. The different combinations were tested in presence and absence of two organohalide molecules, Cl-OHPA or 3,5-DCP. The top panel of the figure shows the gels from which DNA signals were quantified and expressed in the bottom panel (for **B, C**) as free DNA (dark gray) and protein/DNA complex (light gray). The asterisk indicates the position where free DNA migrated in each of the experiments.

Both RdhK hybrids along with RdhK6 and RdhK1 proteins were analyzed by EMSA upon exposure to the effectors Cl-OHPA and 3,5-DCP, and to DB DNA motifs embedded in *E. coli mgl* promoter (Supplementary Figure 1). To serve as negative control, a version of the *mgl* promoter was included which displayed a random and non-palindromic sequence, named noDB. No interaction was observed with this sequence for any of the four RdhK proteins, which confirmed that all observed ternary complexes described in the following sections depend on the presence of a genuine DB motif (Figure 3A). Additionally, a series of experiments was also performed as control with a 1:1 mixture of the two effectors to show that no competition nor inhibition events are responsible for the absence of ternary complex. Globally, the interactions pattern remained similar when both effectors were present (Supplementary Figure 3).

#### *In vitro* Binding of RdhK Hybrids to DB8

According to the design presented above, both hybrids were expected to bind specifically to DB8 in presence of Cl-OHPA. Yet, a first series of experiments was performed using DB8 as target DNA motif (Figure 3B). Upon exposure to Cl-OHPA, nearly 100% of DB8 was retained in a ternary complex by both hybrids. When Cl-OHPA was omitted or replaced by 3,5-DCP, no positive interaction could be observed with Hybrid B. The latter response agreed with what was expected with both hybrid proteins. In contrast, Hybrid A showed a slight binding to DB8 in presence of 3,5-DCP. Since a similar level of protein-DNA complex was also observed without effector, this most probably reflects a constitutive DNA-binding activity of this hybrid protein, which, however, was enhanced in the presence of Cl-OHPA. It was also noticed that the bands corresponding to DB8 in complex with Hybrid A did not appear as resolved as the ternary complexes observed with other proteins. Additionally, residual smears were observed on the top half of the lanes in EMSA experiments with Hybrid A. Consequently, the bands corresponding to protein-free DNA in these lanes were less intense even though the same initial DNA concentration was used for all reactions. These two observations may be explained by a possible aggregation of purified Hybrid A.

#### *In vitro* Binding of Parental RdhK to DB8

As expected, nearly all DB8 was found in complex with RdhK1 when 3,5-DCP was used as effector molecule (Figure 3B). Also, in agreement with earlier work, a significant but lower amount of DB8 was retained in the complex without the addition of any effector (Gábor et al., 2008). The addition of Cl-OHPA did not increase the proportion of DB8 in complex with RdhK1 and indicated that this effector had likely no positive effect on RdhK1 for binding DB8. Gábor et al. proposed that a constitutive activation of the RdhK1 protein could explain these observations and may be considered of physiological relevance. They also suggested that only a ternary complex is able to recruit the RNA polymerase suggesting that effector-free RdhK1 protein can bind to the promoter but may not be sufficient to induce gene transcription (Gábor et al., 2008), as it was also described for other members of the CRP/FNR superfamily (Diaz and Prieto, 2000). Nevertheless, RdhK1 affinity for DB8 was enhanced by the addition of 3,5-DCP. RdhK6 was similarly tested for its interaction with DB8. Since this DNA motif is part of the *rdh-1* gene cluster, this combination has not been tested in earlier work and a positive interaction was unforeseen. However, nearly 100% of DB8 was retained in complex with RdhK6, when and only when Cl-OHPA was added to the reaction. This result suggested a potential crosstalk of RdhK6 with different *rdh* gene clusters, a phenomenon that was not considered so far.

#### *In vitro* Binding of RdhK Hybrids to DB7

A second set of experiments was run with DB7 (Figure 3C). Since DB7 is the DNA motif targeted by RdhK6, the hybrids harboring RdhK1 DBD were not expected to bind this motif. No complex formation was observed with Hybrid B, even upon addition of Cl-OHPA. In contrast, approximately 90% of DB7 was retained in a ternary complex by Hybrid A in presence of Cl-OHPA. This result denoted a rather moderate DNA specificity of Hybrid A. Here again, the band corresponding to the ternary complex appeared less resolved and smears were observed in the top part of all lanes loaded with Hybrid A, as described in the DB8 experiment series. In contrast with the latter, the formation of complexes between Hybrid A and DB7 appeared to be strictly dependent on the presence of Cl-OHPA, as no corresponding bands were observed in its absence. This suggested that the constitutive binding activity of Hybrid A described above is only true for DB8.

#### *In vitro* Binding of Parental RdhK Proteins to DB7

In agreement with previously reported data, RdhK6 was able to retain almost 100% of DB7 when, and only when, Cl-OHPA was added to the reaction (Smidt et al., 2000; Gábor et al., 2006, 2008). In addition, RdhK1 showed a weak binding affinity for DB7, independently of the presence of any effector molecule. Although this recall the observations described with DB8, only 10% of DB7 was retained in complex by RdhK1 (against 80% of DB8, with no effector). Furthermore, unlike in DB8 series, the addition of 3,5-DCP did not enhance RdhK1 affinity for DB7. This suggests that the binding events observed between RdhK1 and DB7 are most probably the result of a residual activity of the protein while DB8 remains its true target motif.

The results presented in this section revealed that the *in vitro* DNA-binding activity of both hybrid proteins was enhanced by the presence of Cl-OHPA, which emphasizes their ability to recognize this compound as effector and supports the production of hybrids actively binding DB motifs by fusing the EBD and DBD originating from different RdhK proteins. The results of EMSA experiments also highlighted that Hybrid A has only a limited DNA-binding specificity whereas Hybrid B is highly specific for the targeted DB as well as the effector. This shows a promising potential of the latter design for the investigation of uncharacterized RdhK proteins.

### *In vivo* Characterization of RdhK Hybrid Proteins

#### Control and Preliminary Experiments

To validate the data obtained *in vitro* and to further evaluate whether the two hybrids can act as genuine transcription activators beyond promoter binding, both hybrids were investigated with the *in vivo* β-galactosidase reporter assay as described earlier (Gábor et al., 2006). Hybrids A and B were expressed in *E. coli* cells carrying a reporter plasmid with the β-galactosidase gene placed under the control of promoters carrying either the DB7, DB8 or noDB DNA motif (Gábor et al., 2006). To focus on the specificity of the DB motifs exclusively, the native promoter region of *cprBA* (harboring DB7 or replaced, when applicable, by DB8 or noDB) from *D. hafniense* strain DCB-2 was used (Supplementary Figure 2). A preliminary *in vivo* reporter assay was first performed in small volumes to screen for positive interactions between RdhK proteins and the DB motifs in presence of Cl-OHPA (Supplementary Figure 4). With noDB, the correlation between the addition of Cl-OHPA and the increase of β-galactosidase activity was either negative or not significant (*p*-value > 0.05). Therefore, this control DNA motif was not further investigated. Experiments involving DB7 and DB8 were then performed in larger culture volumes to improve signal to noise ratio.

#### *In vivo* Binding Affinity of RdhK Hybrids for DB8 and DB7

**FIGURE 4:**
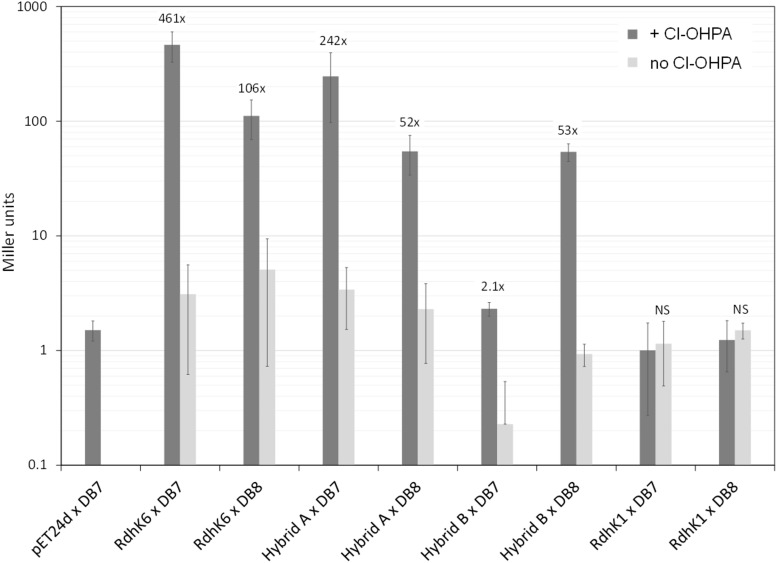
*In vivo* β-galactosidase reporter assay of RdhK parental and hybrid proteins. Each of the four proteins (RdhK6, RdhK1 and Hybrid A and B) was tested for their ability to promote β-galactosidase activity under promoters that display either the DB7 or DB8 motif. The resulting β-galactosidase activity was measured and is expressed in Miller units. Cl-OHPA was added as effector (dark gray bars). A control was included for each RdhK/DB combination, where no effector was added to the culture during protein expression (light gray bars). All experiments were performed in three biological replicates.

Upon addition of Cl-OHPA, a significant increase in β-galactosidase activity of 52- and 53-fold (*p*-value < 0.05) was observed with DB8 for Hybrid A and B, respectively (Figure 4). This result demonstrated that both hybrids can act as transcription regulator and recognize DB8 as target DNA motif. In the absence of Cl-OHPA, the β-galactosidase activity with both hybrids dropped to a level similar to the one obtained with cells carrying the empty pET24d vector instead of the hybrid expression plasmids. This confirmed that the enhancement of the β-galactosidase activity was dependent on the production of the corresponding hybrid proteins. When DB7 was used in combination with Hybrid B, the increase of β-galactosidase activity in presence of Cl-OHPA was only 2-fold, which likely suggested a poor affinity for this DNA motif in comparison to DB8. In contrast, the β-galactosidase activity measured with Hybrid A with DB7 increased about 250-fold upon addition of Cl-OHPA. The increase observed with DB7 was approximately five times more important than with DB8, which may indicate a higher affinity of Hybrid A for DB7 than for DB8. This trend was not observed *in vitro* and thus emphasizes the need of using both *in vitro* and *in vivo* strategies to fully describe the behavior of RdhK hybrid proteins. Globally, the results obtained from *in vivo* experiments with both hybrids were in good agreement with the conclusions drawn from the *in vitro* data. However, one aspect of the Hybrid A *in vitro* response did not have a visible counterpart in the *in vivo* results. Indeed, in the latter, Hybrid A activity remained dependent on the presence of Cl-OHPA at any time. Thus, the constitutive *in vitro* DNA-binding activity proposed for this hybrid is most likely an artifact resulting from the unstable behavior of the purified protein rather than a genuine DNA-protein interaction responsible for gene transcription. Moreover, this observation could also be explained by the fact that a protein-DNA complex in absence of the effector may not be sufficient to properly induce gene transcription.

#### *In vivo* Binding Affinity of Parental RdhK for DB8 and DB7

All the experiments described above were also performed with the two parental RdhK proteins. Despite many trials, no β-galactosidase activity could be measured with RdhK1. This observation is different from what has been described in the work by Gábor et al., in which the activity of RdhK1 was significantly higher when it was combined with DB8 in comparison to other DB motifs (Gábor et al., 2008). An additional set of experiments was performed using 3,5-DCP as effector molecule for RdhK1 but resulted also in no β-galactosidase activity (data not shown). These negative results indicated that the experimental conditions applied here prevented the production of an active form of RdhK1 or that the design of the reporter platform based on *cprBA* intergenic region may not be compatible with RdhK1 recognition of DB8. In contrast, RdhK6 in combination with DB7 gave the highest β-galactosidase activity with a significant increase of 461-fold upon addition of Cl-OHPA. Also, in agreement with the *in vitro* results, an increase in activity of 106-fold was measured when RdhK6 was combined with DB8. This result indicates again a potential physiological crosstalk between two *rdh* gene clusters in *D. hafniense* strain DCB-2.

## Discussion

### RdhK Hybrid Proteins Are Active Regulatory Proteins

In this study, we show that the two domains (EBD and DBD) coming from different native RdhK proteins can be fused and the resulting hybrid proteins can be produced heterologously in *E. coli* as soluble and active proteins. Even though the results obtained with Hybrid A and Hybrid B are different in their binding properties, both proteins were active *in vitro* as well as *in vivo*. As expected, the hybrid proteins showed binding affinity for the DNA motif DB8 (initial target of RdhK1) which was enhanced in presence of Cl-OHPA (effector molecule of RdhK6). Thus, both hybrid designs proposed in this work gave rise to actual transcription regulators with binding properties that were neither equivalent to RdhK6 nor RdhK1. One aspect that Hybrid A and B had in common was the relatively low β-galactosidase activity. Indeed, the values obtained upon addition of Cl-OHPA for the hybrid proteins in combination to DB8 were always significant but remained relatively low in comparison to the activity measured with RdhK6 and DB7 (a mean value of 465 Miller units for RdhK6/DB7 against 55 for the hybrids/DB8). The RdhK6 activity is in good agreement with former reports (Pop et al., 2004; Gábor et al., 2006, 2008), which indicates that the experimental set-up is likely not the reason for the lower activity measured with both hybrids. Pop et al. reported a higher activity upon increasing the final concentration of Cl-OHPA in the cultures (Pop et al., 2004). Here, the addition of 20 mM Cl-OHPA did not result in an increase of the activity for the hybrid proteins (data not shown). A reduced expression rate or a reduced solubility of the hybrid proteins compared to RdhK6 might be the reason for the difference in activity. However, under the same experimental conditions, the unforeseen activity obtained with Hybrid A in combination with DB7 was much higher than with DB8 (a mean value of 246 Miller units) suggesting that an impaired protein expression may not be the only reason for the reduced activity. The values reported by Gabor et al. for their experiments involving RdhK1 and DB8 are in the same range as the ones we obtained with the hybrid proteins in combination with DB8 embedded in the *cprBA* promoter region (Gábor et al., 2008). Unfortunately, we could not confirm the values for RdhK1 activity but the fact that, globally, all the results obtained with DB8 gave lower activity (in absolute terms) suggests that the promoter sequence beyond the DB motif may also play a role. Moreover, DB8, unlike DB7, is only partially palindromic, therefore possibly explaining the lower activity.

### Hybrid B Is More Specific Than Hybrid A

The comparison of the two hybrid proteins in the *in vivo* and *in vitro* approaches revealed a higher DNA-binding specificity of Hybrid B over Hybrid A, as the latter recognized both DB8 and DB7. The *in vivo* experiments even showed a higher response when Hybrid A was combined with DB7 and may indicate a higher affinity for this motif. In addition, Hybrid A gave an unclear *in vitro* response that can likely be explained by a limited protein stability upon purification. Only Hybrid B showed a consistent ON/OFF response upon addition of Cl-OHPA, both *in vitro* and *in vivo*. The fact that Hybrid A is less specific in DNA-binding than Hybrid B remains difficult to explain since the former protein has a higher portion of RdhK1 C-terminal domain than the latter and should, consequently, be as stringent for DNA motifs as the parental RdhK1 protein if only the DBD is responsible for DNA recognition. This suggests that the protein-DNA interaction involves other residues than initially thought and may not be after all restricted to the very last region of the C-terminus. However, both hybrid proteins were able to recognize Cl-OHPA as effector, while 3,5-DCP did not activate them. Despite the fact that Hybrid A has a shorter portion of RdhK6 EBD, both hybrid proteins display all the residues that have been previously reported to be important for the interaction with Cl-OHPA (Joyce et al., 2006; Levy et al., 2008; Kemp et al., 2013). Furthermore, since RdhK1 and RdhK6 are relatively close at the amino acid sequence level (89% identity), many of the residues highlighted in previous studies are essentially conserved in the two RdhK proteins and therefore also in the two hybrid proteins. The global conformation or the stability of both Hybrids may be partially responsible for the distinct binding patterns. Probably only a structural approach would allow us to clarify why Hybrid A is less specific in DNA binding. Nevertheless, our results showed that the strategy of domain fusion in Hybrid B created a higher specificity for the effector and for DNA than that of both parental proteins. Indeed, Hybrid B showed a higher effector dependency than RdhK1 and a higher DNA specificity than RdhK6.

### RdhK6 Recognizes DB Motifs Located in Other Gene Clusters

Beside the results obtained from the RdhK hybrid proteins, the different experiments performed in the context of this study revealed an interesting aspect of RdhK6. As expected, RdhK6 showed a high affinity for DB7 in presence of Cl-OHPA, confirming previously reported results (Smidt et al., 2000; Pop et al., 2004; Gábor et al., 2006, 2008; Joyce et al., 2006; Levy et al., 2008). However, our dataset also highlighted the binding of RdhK6 to DB8 both *in vitro* and *in vivo*. In the latter case, Cl-OHPA-induced expression of the β-galactosidase was significantly lower for DB8 than for DB7, which may indicate a lower affinity of the protein for this DB motif and/or a weaker RNA polymerase activity when the DB8 motif replaced DB7 in the native promoter sequence. In any case, this observation points toward a new feature of RdhK proteins that has not been investigated in earlier work. Indeed, as DB8 is located in the *rdh-1* gene cluster (harboring *rdhK1*), this result suggests a possible crosstalk of RdhK proteins with the different *rdh* gene clusters. Comparing the results of the present study with earlier studies, it appears that RdhK6 has affinity for all the DB tested so far, which include the three DB sequences identified in its own gene cluster (Gábor et al., 2008), the modified version of the FNR box (Gábor et al., 2006) and DB8 (this study). The alignment of these selected DB motifs shows that only the first two nucleotides (and the corresponding last two positions) are fully conserved in the palindrome (Supplementary Figure 5), thus suggesting that RdhK6 regulation network might be broader than initially thought.

## Conclusion

The present work shows the potential of using RdhK hybrid proteins to screen for DNA motifs targeted by yet uncharacterized RdhK proteins. Two different hybrid proteins were successfully produced in an active form and gave distinct response by *in vitro* and *in vivo* characterization. While Hybrid A showed a mixed behavior between the constitutive activation of RdhK1 and the relaxed DNA motif specificity of RdhK6, Hybrid B displayed a reliable and specific response both *in vitro* and *in vivo*. The latter appears as the design of choice to serve as a basis to create new RdhK hybrids for the investigation of uncharacterized RdhK DNA-binding domains by fusing them to the effector- binding domain of RdhK6. The proposed strategy based on the design of RdhK6_*E*_ hybrid proteins could be applied to investigate the OHR regulation network in *Dehalobacter restrictus* strain PER-K23. This strain harbors up to 22 members of the RdhK protein subfamily, but is so far only known to dechlorinate tetra- and trichloroethene. Given the fact that many families of transcription regulators belong to the superfamily of CRP/FNR proteins (Matsui et al., 2013), this strategy could provide a tool for the screening of DNA motifs for virtually any CRP/FNR protein.

## Data Availability Statement

All datasets generated for this study are included in the article/Supplementary Material.

## Author Contributions

JM initially designed the project. MW and JM refined the global strategy and wrote the manuscript. MW performed all the experimental assays. MV technically supported MW in many of the experiments and constructed most of the plasmids. CH revised the manuscript.

## Conflict of Interest

The authors declare that the research was conducted in the absence of any commercial or financial relationships that could be construed as a potential conflict of interest.
