# Supplementary material for: Hybrid Transcriptional Regulators for the Screening of Target DNA Motifs in Organohalide-Respiring Bacteria
Source: Front Microbiol. 2020 Mar 3;11:310. doi: 10.3389/fmicb.2020.00310 (PMC7062800; doi:10.3389/fmicb.2020.00310)
Supplement: Supplementary file 1 [file Data_Sheet_1.PDF]

Supplementary Material

Hybrid transcriptional regulators for the screening of target DNA motifs in organohalide-respiring bacteria

Mathilde Stéphanie Willemin, Marie Vingerhoets, Christof Holliger and Julien Maillard*

Correspondence: julien.maillard@epfl.ch

1 Supplementary Data

1.1 Fusion PCR

Two primer pairs were used in Fusion PCR: F1, R1, F2 and R2. Primers F1 and R2 were designed as primers targeting the beginning and the end of the overall sequence to amplify. When needed, they include a restriction site to subsequently insert the sequence in the expression plasmid. Primers R1 and F2 are targeting the end of the first domain and the beginning of the second domain, respectively. They include a small tail of 5 nucleotides complementary to the fusion site. In a first round of PCR, the coding sequence of the two domains were amplified separately using the pair of primers F1/R1 and F2/R2, respectively. The two PCR products are then mixed in a 1:1 ratio (typically 50 ng of each products were completed with ddH_2O to a total of 2 µL) to serve as template for a second round of PCR with primers F1 and R2 to generate the final PCR product.

Reactant	Stock Concentration	Round-1		Round-2 Fusion PCR
		1st domain	2nd domain	
Hi-Fi Buffer*	5×	10 µL	10 µL	10 µL
DMSO	100 %	1.5 µL	1.5 µL	-
$MgCl_2$	50 mM	2 µL	2 µL	2 µL
dNTPs	10 mM	5 µL	5 µL	5 µL
Genomic DNA	>5 ng/µL	2 µL	2 µL	-
Mix of 1st and 2nd domains	50 ng each	-	-	2 µL
Primer F1	10 µM	2.5 µL	-	1.25 µL
Primer R1	10 µM	2.5 µL	-	-
Primer F2	10 µM	-	2.5 µL	-
Primer R2	10 µM	-	2.5 µL	1.25 µL
ddH_2O	100 %	23.5 uL	23.5 uL	27.5 µL
VELOCITY DNA Polymerase*	-	1 µL	1 µL	1 µL
Final volume	-	50 µL	50 µL	50 µL

(*Bioline, Labgene scientific, Châtel-St-Denis, Switzerland)

2 Supplementary Table

Table 1. List of oligonucleotides used in the study.

Primer	Sense	Sequence (5'→3')	Gene target / plasmid	Reference
MW031	+	GAATCCATGGGAGAAATTCTT AAAAATTATATTTTTC	*rdhK1* / pET24d	This study
MW032	-	GCGCAAGCTTTCAATAAGCTA TCCCCTCTTCGAGAAG	*rdhK1* / pET24d	This study
RdhK61A-F	+	GAATCCATGGGCGTTGAGGGG TTAGGAAAAGAC	*rdhK61A* / pET24d	This study
RdhK61A-R	-	GCGCAAGCTTTCAATACGCAA TGCCTTCTTCCAG	*rdhK61A* / pET24d	This study
MW033	+	GAATCCATGGCTGTTGAAGGT TTGGG	*rdhK61B* (EBD) / pET24d	This study
MW034	-	TGAAGACCGGTAATCTCCCCA ATACTCTT	*rdhK61B* (EBD) / pET24d	This study
MW035	+	CCGGTCTTCATTATGTCACCGT AAGCAAGATA	*rdhK61B* (DBD) / pET24d	This study
MW036	-	GCGCCTCGAGTCAATAAGCTA TCCCCTCTTCGAGAAG	*rdhK61B* (DBD) / pET24d	This study
DBC-F	+	GAATTCTCAGGCGATGTAAC	*mgl*-DB chassis	This study
DBC-R	-	CGGTGCAATCATAGCTATC	*mgl*-DB chassis	This study
mgl-DB7	+	GAATTCTCAGGCGATGTAACC GCTTTCAAAGTTAATACACAT TAATACTCTTAACAATGTGAT AGCTATGATTGCACCG	DB7 internal primer / *mgl*-DB chassis	This study
mgl-DB8	+	GAATTCTCAGGCGATGTAACC GCTTTCAAATTTAGTATACGCT AAATATCTTAACAATGTGATA GCTATGATTGCACCG	DB8 internal primer / *mgl*-DB chassis	This study
mgl-noDB	+	GAATTCTCAGGCGATGTAACC GCTTTCAGCCTCTAGTGGCTG GCTTGATCTTAACAATGTGAT AGCTATGATTGCACCG	noDB internal primer / *mgl*-DB chassis	This study
BG1743	+	GCGCAAGCTTGGCCAAGGTCG CCATGACG	*cprBA* promoter / pAK80	(Gabór, 2006)
BG1704	-	GCGGATCCGCTATAAAAACTA AAAATGTACC	*cprBA* promoter / pAK80	(Gabór, 2006)
MW060	-	TCTCAACCTTTCCTTCCGTAGT	*cpr*-DB7 promoter / pAK80	This study
MW061	+	GATCCTTATTACAATATACCC ATACTACTTT	*cpr*-DB7 promoter / pAK80	This study
MW062	+	TGAGAAATTCAGGTAAATTTA GTATACGCTAAATATTGCGTA TGTGATCC	*cpr*-DB8 internal primer / pAK80	This study
MW063	+	TGAGAAATTCAGGTAGCCTCT AGTGGCTGGCTTGATTGCGTA TGTGATCC	*cpr*-noDB internal primer / pAK80	This study

3 Supplementary Figures

(A)

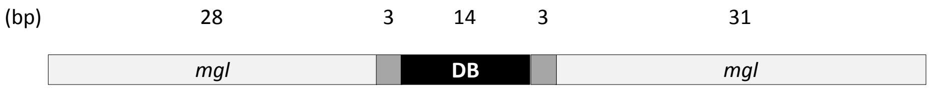

(B)

P_{mgl}
AACAAATCAGGCGATGTAACCGCTTTCAATC**TGTGA**GTGATT**TCACA**GTATCTTAACAATGTGATAGCTATGATTGCACCG
-CRP-......-CRP- .-10.. +1

***mgl*-DB7**
*gaattctcaggcgatgtaaccgctttca*AAG**TTAAT**ACAC**ATTAA**TAC*tcttaacaatgtgatagctatgattgcaccg*

***mgl*-DB8**
*gaattctcaggcgatgtaaccgctttca*AAT**TTAGT**ATAC**GCTAA**ATA*tcttaacaatgtgatagctatgattgcaccg*

***mgl*-noDB**
*gaattctcaggcgatgtaaccgctttca*GCCTCTAGTGGCTGGCTTGA*tcttaacaatgtgatagctatgattgcaccg*

Supplementary Figure 1. Design of *mgl*-DB chassis for EMSA analysis. **(A)** Schematic representation of the *mgl* promoter containing the DB motif. Each *mgl*-DB DNA fragment is composed by a unique 20-nt long sequence corresponding to the DB sequence (14 nt) flanked by 3 nt on each side from the original *rdh* promoter, and embedded in a common DNA chassis derived from the *mglBAC* promoter (P_{mgl}) from *E. coli*. This design was chosen to avoid experimental incompatibility as the *mgl* promoter shares structural features with promoters targeted by RdhK proteins and is known to be the target of CRP (Zheng et al. 2004). **(B)** For each DB of interest, a 79-nt long oligonucleotide was designed and used as template for PCR amplification with a pair of primers targeting the 5'- and 3'-ends of P_{mgl}.

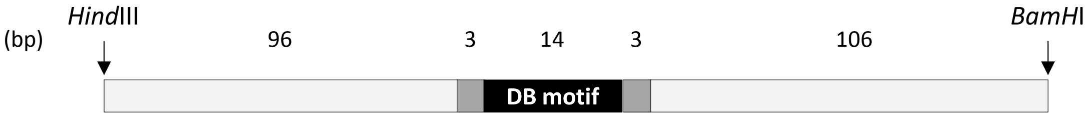

***cprBA-based* DB chassis**

AAGCTTGGCCAAGGTCGCCATGACGGCTCTGCGCAATGCTCTGGGCATTGTGTCCATTATGCTGACTACGGAAGG
AAAGGT***TGAGAAATTCAGGTA-20-nucleotides-TTGCGTATGTGATCC***TTATTACAATATACCCATACTAC
TTTTTTATTATCTTGCTAAAGGGAGGTGAAAAAGTTATGGGTACATTTTTAGTTTTTATAGCGGATCC

DB7 AAG**TTAAT**ACAC**ATTAA**TAC
DB8 AAT**TTAGT**ATAC**GCTAA**ATA
noDB GCCTCTAGTGGCTGGCTTGA

Supplementary Figure 2. Design of the *cprBA*-based DB chassis for *in vivo* β-galactosidase reporter assay. Schematic representation of the cloning strategy for the DB chassis used in fusion with the reporter gene. The DB7 motif and three flanking nucleotides on each side (20 bp in total) was replaced by DB8 or the non-palindromic noDB sequence. Using DB motifs embedded in the native *cprBA* promoter was chosen to address the question of the specificity of the DB motif exclusively. The 222-bp fragments were produced by fusion PCR (see Supplementary Data for details), and cloned into pAK80 carrying the β-galactosidase gene.

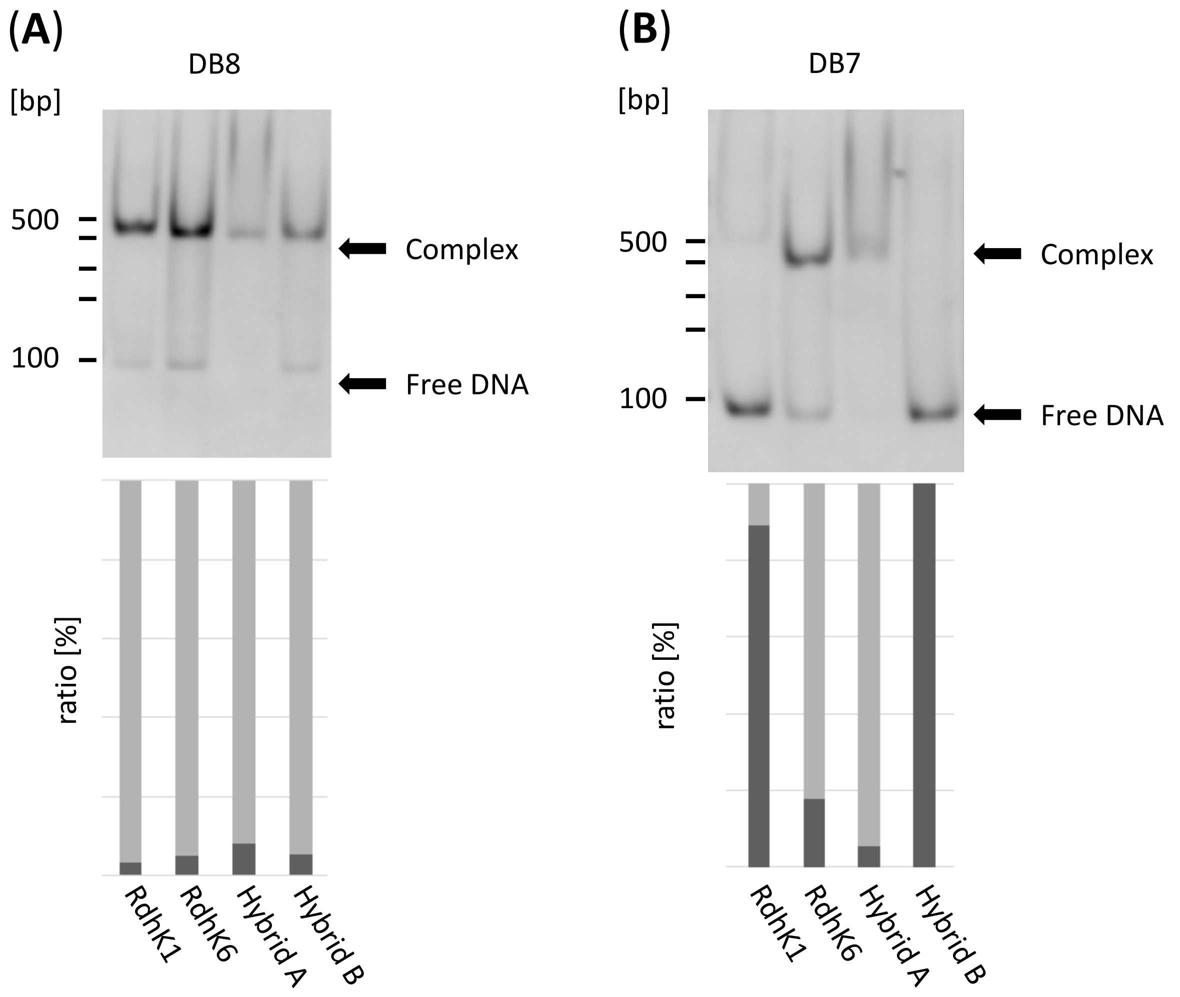

Supplementary Figure 3. Control EMSA experiments with the four RdhK proteins, DB8 (**A**) or DB7 (**B**), and the combination of both effectors. Both effectors (Cl-OHPA and 3,5-DCP) were added in a 1:1 ratio in the reactions. This experiment was used to exclude the influence of potential inhibitory or competitive events between the two compounds.

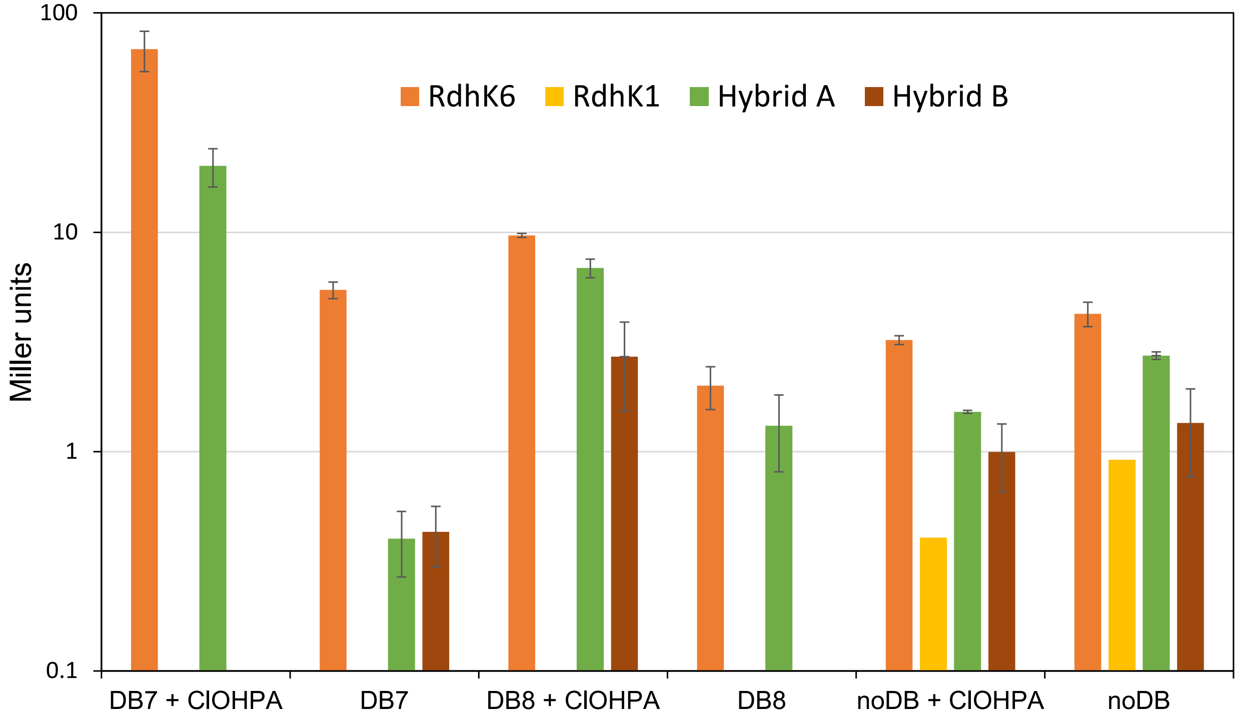

Supplementary Figure 4. Preliminary *in vivo* β–galactosidase assay for protomer-binding activity of RdhK proteins. The preliminary experiment was performed at small scale with all four proteins (RdhK6, RdhK1, Hybrid A and B) in combination with promoters carrying either DB7, DB8 or noDB motif in presence/absence of Cl-OHPA as effector. Each experiment was run in triplicates and the protocol was adapted to be run in a screening mode using a 96-well reader plate. The most interesting RdhK/DB combinations were further tested at a larger scale in order to strengthen the signal-to-noise ratio.

```
DB5   TTATTgcacATTAA
DB6   TTAGTgcacTCTAA
DB7   TTAATacacATTAA
DB8   TTAGTatacGCTAA
FNR   TTGATacacATCAA
```

Supplementary Figure 5. Sequence alignment of DB motifs recognized by RdhK6. The DB5, DB6 and DB7 motifs are part of the *rdh-6* gene cluster, DB8 belongs to the *rdh-1* gene cluster, while FNR motif represents the paradigmatic palindrome recognized by FNR.
